# Cardiovascular Mortality and Leaded Aviation Fuel: Evidence from Piston-Engine Air Traffic in North Carolina

**DOI:** 10.3390/ijerph19105941

**Published:** 2022-05-13

**Authors:** Heather Klemick, Dennis Guignet, Linda T. Bui, Ron Shadbegian, Cameron Milani

**Affiliations:** 1 National Center for Environmental Economics, US Environmental Protection Agency, Washington, DC 20460, USA; 2 Department of Economics, Walker College of Business, Appalachian State University, Boone, NC 28608, USA; guignetdb@appstate.edu; 3 Department of Economics, International Business School, Brandeis University, Waltham, MA 02453, USA; ltbui@brandeis.edu; 4 Department of Economics, College of Arts and Letters, San Diego State University, San Diego, CA 92182, USA; rshadbegian@sdsu.edu; 5 Department of Politics and Economics, Claremont Graduate University, Claremont, CA 91711, USA; cmilani19@gmail.com

**Keywords:** lead exposure, aviation fuel, air pollution, mortality, cardiovascular, elderly, epidemiology, Q53, I18

## Abstract

Leaded fuel used by piston-engine aircraft is the largest source of airborne lead emissions in the United States. Previous studies have found higher blood lead levels in children living near airports where leaded aviation fuel is used. However, little is known about the health effects on adults. This study is the first to examine the association between exposure to aircraft operations that use leaded aviation fuel and adult cardiovascular mortality. We estimated the association between annual piston-engine air traffic and cardiovascular mortality among adults age 65 and older near 40 North Carolina airports during 2000 to 2017. We used several strategies to minimize the potential for bias due to omitted variables and confounding from other health hazards at airports, including coarsened exact matching, location-specific intercepts, and adjustment for jet-engine and other air traffic that does not use leaded fuel. Our findings are mixed but suggestive of adverse effects. We found higher rates of cardiovascular mortality within a few kilometers downwind of single- and multi-runway airports, though these results are not always statistically significant. We also found significantly higher cardiovascular mortality rates within a few kilometers and downwind of single-runway airports in years with more piston-engine air traffic. We did not consistently find a statistically significant association between cardiovascular mortality rates and piston-engine air traffic near multi-runway airports, where there was greater uncertainty in our measure of the distance between populations and aviation exposures. These results suggest that (i) reducing lead emissions from aviation could yield health benefits for adults, and (ii) more refined data are needed to obtain more precise estimates of these benefits. Subject Areas: Toxic Substances, Health, Epidemiology, Air Pollution, Ambient Air Quality. JEL codes: Q53, I18.

## 1. Introduction

Lead is a neurotoxin that damages multiple systems in the body. While neurodevelopmental effects in children are well documented, adults are also adversely affected by lead exposure [1]. Of particular concern is the increased risk of cardiovascular morbidity and mortality in adults [1,2].

Piston-engine aircraft operations using leaded fuel (termed aviation gasoline, or avgas) currently represent the largest source of airborne lead in the U.S., contributing 70% of lead emissions [3]. While jet-engine aircraft (which do not use leaded fuel) dominate commercial air traffic, smaller piston-engine aircraft are widely used for non-commercial purposes that fall under the heading of general aviation, including commuting, recreation, flight instruction, and agriculture. There are roughly 13,000 airports nationwide, most of which include piston-engine airplane traffic [4].

The United States Environmental Protection Agency (EPA) estimates that over 5 million people live in census blocks located within 500 m of a runway at an airport with piston-engine aircraft [5]. Previous studies have found an association between exposure to leaded aviation fuel and children’s blood lead levels (BLLs) [6,7,8]. There is also a growing literature examining the association between lead exposure and adult cardiovascular mortality [9,10,11,12,13]. However, to our knowledge, no studies have examined how exposure to leaded aviation fuel emissions affects cardiovascular mortality.

We addressed this gap in the literature by estimating the effects of proximity to airports and of year-to-year changes in piston-engine and general aviation aircraft operations on cardiovascular mortality rates among older adults from 2000 to 2017 in North Carolina. Our study used a quasi-experimental research design that examined the association between piston-engine operations and annual cardiovascular mortality rates among individuals age 65 and older living in census block groups closer to airports (the “treated” group) and farther away from airports (the “control” group). We used coarsened exact matching [14] to ensure that our treated and control groups were similar in terms of observable socioeconomic characteristics that could affect cardiovascular mortality. We addressed the potential for confounding of leaded fuel exposure with other health hazards at airports by controlling for different types of aircraft operations that do not emit lead but do generate noise and other pollutants such as particulate matter and volatile organic compounds associated with cardiovascular disease. We included block group intercepts to control for unobserved determinants of cardiovascular mortality at the neighborhood level that remain stable over time. We also examined how these associations varied with location downwind and upwind of airport runways.

Our findings were mixed. We found higher cardiovascular mortality rates within a few kilometers downwind of single- and multi-runway airports, though these results were not always statistically significant. We also found higher cardiovascular mortality rates near single-runway airports in years with more piston-engine air traffic. The magnitude of this association declined monotonically from 1 km to 3 km from airport runways. This adverse effect was even more pronounced within 1 km downwind of single-runway airports. However, we did not find consistent statistically significant adverse effects from changes in annual piston-engine operations near multi-runway airports, where there was greater uncertainty about which runways and block groups experienced exposures to leaded fuel emissions. The statistically significant adverse effects were also limited to Instrument Flight Rules (IFR) piston-engine flights, which are explicitly tracked by FAA computer systems. We did not find statistically significant adverse effects for general aviation flights for which the FAA data were less reliable.

## 2. Background on Leaded Aviation Gasoline

Tetraethyl lead is added to aviation gasoline to boost octane and prevent engine knock. Since the 1970s, piston-engine aircraft have predominantly used a grade of avgas called one hundred octane low lead (100LL) containing 2.12 g of lead per gallon [15]. Alternatives with lower lead levels are not widely available, and unleaded fuels that meet the octane requirements for high-performance piston-engine aircraft have not yet been developed [15]. Piston-engine helicopters, which are not the focus of our study, also use leaded fuel. (Piston-engine helicopters comprise two percent of the approximately 144,000 piston-engine aircraft currently active in the U.S. and account for four percent of hours flown [15]. The EPA’s limited characterization of lead emissions from helicopters suggested that they generate lower air lead concentrations than fixed-wing airplanes, particularly during takeoff and landing [4]). In contrast, jets, military planes, and other turbine-engine aircraft use unleaded fuel.

Piston-engine aircraft lead emissions occur throughout the phases of a flight, including start-up, idling, taxiing, run-up, takeoff, cruising, and landing. Lead emissions are highly concentrated during ground-based run-up operations conducted prior to takeoff, next to the end of the runway, making this area the maximum impact site for lead air concentrations at airports [4]. Releases also occur in maintenance and refueling areas. Piston-engine airplanes typically take off in the direction of the wind, so the maximum impact site can change with wind direction, particularly at airports with more than one runway.

EPA has discussed considerations in the determination of whether aircraft lead emissions endanger public health or welfare but has not yet issued a proposed determination evaluating endangerment [16]. In 2008 and 2010, EPA established new monitoring requirements for sources emitting lead. As a result, state and local agencies were required to monitor lead at airports where emissions estimates exceeded one ton per year and at a subset of airports that met certain criteria [17]. The three-month average lead concentrations at the airport monitors ranged from 0.01 to 0.33 µg/m^3^, with the range in concentrations largely explained by monitor location relative to the end of the runway. Monitoring values exceeded the National Ambient Air Quality Standard (NAAQS) for lead of 0.15 µg/m^3^ over a rolling three-month average at two of the airports. In 2020, the EPA extrapolated air quality modeling results to estimate three-month average lead concentrations at U.S. airports nationwide and found that they ranged from 0.0075 µg/m^3^ to 0.475 µg/m^3^ at the maximum impact site and up to 500 m downwind [4]. In most cases, values were not estimated to exceed the NAAQS. However, modeling showed that it is possible for levels to exceed the NAAQS at the maximum impact site at airports with relatively high numbers of piston-engine aircraft operations, particularly those with a higher proportion of multi-engine aircraft. In early 2022, the EPA announced plans to issue a proposed endangerment finding.

## 3. Literature Review

Previous studies have examined the effect of exposure to aviation fuel on children’s blood lead levels. A study of six counties in North Carolina found higher BLLs among children living within 1.5 km of airport boundaries after controlling for other lead exposure risk factors including socioeconomic status and housing age [6]. A study in Michigan found higher BLLs among children in census tracts up to 3 km from airports and up to 4 km from airports for which data on monthly aviation traffic were available from the Federal Aviation Administration (FAA) [7]. That study also found higher BLLs downwind of airports and during months with more piston-engine air traffic. In addition, Zahran et al. found a drop in BLLs corresponding to the grounding of air traffic after the 11 September 2001, terrorist attacks. A recent report examining an airport in Santa Clara County, California, found higher children’s BLLs closer to the airport, downwind of the airport, and in months with more piston-engine air traffic [8]. Wolfe et al. did not conduct an empirical analysis of children’s BLL but instead used air quality modeling and existing statistical relationships between air lead concentrations, blood lead levels, and children’s IQ to estimate the social costs of leaded avgas emissions [18]. They estimated that aircraft-related emissions cause over USD 1 billion in losses annually due to cognitive damages that reduce children’s lifetime earnings.

We are aware of only one peer-reviewed study examining occupational exposure to lead from avgas. A study of aircraft maintenance workers in the Republic of Korea found significantly higher BLLs among maintenance crews at air bases where leaded avgas was used compared to air bases where jet fuel was used [19]. Workers’ BLLs also increased with time spent near runways where avgas was used. (A gray literature report on an investigation of potential lead exposures at an aircraft repair and flight school facility found that workers’ BLLs did not exceed 10 micrograms per deciliter, the CDC “level of concern” at that time, nor did air lead levels exceed occupational exposure limits [20]. The investigation did not assess whether worker BLLs were significantly higher than those of the general population of adults).

Empirical studies have also shown increases in air and soil lead levels near airports. Carr et al.’s study of Santa Monica Airport found air lead concentrations above background levels within 450 m of the airport boundaries when averaging over a rolling three-month period and up to 900 m downwind of the airport on individual days [21]. A study of soil lead concentrations near three Oklahoma airports found elevated lead levels near refueling stations, runways, taxiways, and at downwind locations [22]. Higher soil lead levels were also found near the two single-runway airports, possibly because emissions may have been less dispersed than at the multi-runway airport.

The EPA’s Integrated Science Assessment for Lead found robust evidence of a causal relationship between lead exposure and coronary heart disease and hypertension in adults [1]. Lead affects cardiovascular function through multiple mechanisms, including increased oxidative stress, endothelial dysfunction, atherosclerosis, and hypertension, as well as decreased heart rate variability [23]. Several studies have shown a statistically significant relationship between adult BLLs and cardiovascular mortality in U.S. populations with mean BLLs < 5 µg/dL, while controlling for other risk factors including age, sex, race, body mass index, and smoking [9,11,12,13]. A systematic review and meta-analysis found that lead exposure was associated with significantly higher relative risks of cardiovascular disease, coronary heart disease, and stroke [10]. This literature has not examined the sources of lead exposure, though these cohorts were likely to have been exposed to high levels of ambient lead in air prior to the phaseout of lead in road gasoline.

The EPA noted that there is uncertainty about the timing, frequency, and duration of lead exposure causing adverse cardiovascular effects [1]. Because adult BLLs reflect a combination of recent lead exposure and past exposure due to endogenous release of lead stored in bone, the studies mentioned above did not disentangle the contributions of contemporaneous versus past exposures to adverse health effects. Recent evidence, however, suggests that reductions in adult lead exposure can lead to near-term improvements in cardiovascular outcomes. A national-level study of the 2007 voluntary phaseout of leaded gasoline in U.S. auto racing found an immediate decline in annual cardiovascular mortality among those age 65 and older in counties with a racetrack compared to counties without a racetrack [24]. A clinical trial of chelation therapy to remove lead and other heavy metals from patients with severe cardiovascular morbidity caused rapid improvements in cardiovascular function [23].

While we are unaware of existing research on the impact of leaded aviation fuel on cardiovascular health, several studies have examined the health effects of aviation noise and other pollutants. A study of 89 major U.S. airports found that hospitalization for cardiovascular disease was significantly associated with modeled zip-code aircraft noise [25]. Studies in Europe have reported associations between aviation noise and adverse cardiovascular effects [26]. A literature review found adverse health effects in occupationally exposed and residential populations near airports [27]. A study of residential populations within 10 km of California’s 12 largest airports found a significant contemporaneous increase in respiratory and heart-related hospital admissions among those age 65 and older from aviation-related carbon monoxide exposure [28]. Elevated concentrations of fine and ultrafine particulates and other criteria pollutants have been found in residential areas and downwind areas up to several kilometers from major airports [29,30,31].

Studies have also examined whether populations living near airports have different sociodemographic characteristics than those living farther away. EPA’s analysis of populations living within 500 m of airports nationwide found, on average, a higher proportion of White residents, a lower proportion of residents of color, and a slightly lower proportion of children eligible for free or reduced-price lunch near airports compared to the total U.S. population [5]. However, a study of major airport hubs found larger increases in the proportions of residents of color and rental housing units near these airports over time compared to trends in their respective metropolitan regions [32]. A working paper on residential property markets near airports with piston-engine air traffic found that neighborhoods immediately downwind had lower median incomes and a higher proportion of Black residents than other neighborhoods near these airports [33] (That study did not find evidence that property prices changed in response to information disclosures about lead emissions at airports, except for temporary effects at two airports where lead levels exceeded the NAAQS). Thus, previous literature suggests that it is important to address potential confounding from other airport disamenities and to control for neighborhood sociodemographic trends to identify the effect of leaded aviation fuel on cardiovascular outcomes.

## 4. Data

We compiled a comprehensive statewide panel dataset of cardiovascular mortality rates in North Carolina from 2000 to 2017. Like other studies [24,28], we focused on mortality among individuals 65 and older because most cardiovascular mortality occurs within this age group. (Individuals age 65 and older comprise 80% of cardiovascular deaths in the North Carolina 2000–2017 mortality registry). The use of the 18-year time series lends statistical power to our study by increasing both the sample size and the variation in the key explanatory variables, relative to examining a shorter time window.

The unit of observation was each 2010 census block group in each year. Mortality records from the North Carolina State Center for Health Statistics were obtained through an agreement with the Children’s Environmental Health Initiative (CEHI) at the University of Notre Dame. The analysis was conducted according to a research protocol approved by the University of Notre Dame’s Institutional Review Board. We used individual mortality records from North Carolina from 2000 to 2017 (*n* = 1,436,194). The mortality records included the individual’s date of birth, date of death, residential address at the time of death, sex, race, and cause of death as indicated by ICD-10 codes. CEHI used residential address to geocode each record and spatially link it with the corresponding 2010 census block group identifier. We dropped records for individuals not living in North Carolina at the time of death, records not matched to a census block group, and duplicate records (134,003 observations). Because this study focused on cardiovascular mortality among older adults, we further restricted the sample to individuals age 65 or older at the time of death with a disease of the circulatory system listed as the primary cause of death (ICD-10 codes I00-I99) (*n* = 321,445). (Our use of ICD-10 codes I00–I99 is consistent with analyses of the association between adult BLL and cardiovascular mortality [9,11,12]. The most common causes of death were ischemic heart diseases (I20–I25), other forms of heart disease (I30–I52), and cerebrovascular diseases (I60–I69) (see Table S1, Supplementary Materials)).

The Federal Aviation Administration (FAA) provided data on location and aviation traffic for North Carolina airports from a variety of sources (Table 1). We obtained the geographic coordinates of airport runways from FAA Airport Master Records (also called 5010 forms). (FAA shared Airport Master Records for the years 1998 through 2019 at the authors’ request. We obtained data for 2020 online [34]. Geographic coordinates correspond to the Airport Reference Point, a calculation based on the airport runway(s) geodetics (Doug Sage, pers. comm. November 2020)). These data indicated that there were over 400 airports operating in North Carolina during the study period, in addition to heliports and other aviation facilities. Airport Master Records also included data on the number of general aviation single- and multi-engine aircraft based at the airport and the number of operations (i.e., takeoffs or landings) of different flight user classes, including commercial air traffic (air carrier and air taxi), general aviation, and military. However, fewer than half of the airports reported operations data, and those that did report were not required to update the data annually, so the operations data were not always current at the time of reporting. (Out of 435 North Carolina airports for which we had 5010 reports, only 178 reported general aviation operations. At 82 of these airports (46%), reported general aviation operations were the same in every year, suggesting that they may have never been updated. For 5010 forms from 2010 on, we had data on the 12-month period that the reported operations data represented. These data indicated that there was, on average, a two-year lag between the year of the 5010 form and the year the operations data corresponded to).

The FAA Traffic Flow Management System Count (TFMSC) and Air Traffic Activity Data System (ATADS) databases provide more detailed aviation traffic data but for fewer airports [35,36,37]. TFMSC provides daily aircraft operations data by engine type for flights that use Instrument Flight Rules (IFR) and are recorded in FAA’s computer system. These data include operations for approximately 2000 of the largest airports in the United States. TFMSC excludes traffic that flies under Visual Flight Rules (VFR) and some low-altitude IFR traffic. The TFMSC database includes IFR flight records for 72 North Carolina unique airport locations (one of which changed Location Identifiers during the study period). The engine type data provided by TFMSC were particularly useful for our study because only piston-engine aircraft use leaded fuel. Studies of children’s blood lead levels and property values near airports have used TFMSC data [7,8,33].

ATADS includes operations data for approximately 500 US airports with air traffic control towers. ATADS includes both IFR and VFR operations, making it a more comprehensive data source than TFMSC in terms of number of operations. However, it does not provide the engine type; instead, it categorizes operations by user class (air carrier, air taxi, general aviation, and military). (Air carrier and air taxi are both types of commercial operations, with air taxi operations using smaller planes and making shorter trips than air carrier operations. General aviation is defined as all civilian, non-commercial aviation activity). The EPA estimated that roughly 70% of general aviation and 20% of air taxi operations used piston-engine aircraft and, hence, leaded fuel [4]. The ATADS data indicated that VFR flights comprised close to half of general aviation air traffic at these airports. ATADS only included 11 North Carolina airports, all of which were also included in TFMSC. The EPA has used both ATADS and 5010 data to develop estimates of lead emissions and ambient air concentrations from piston-engine air traffic [4].

For each airport with TFMSC data, we obtained the number of IFR departures and arrivals at each airport for each calendar year during 2000–2017 by aircraft engine type (piston, jet, and turbine) and size (small equipment and all larger equipment types). Piston engine flights were of primary interest in our study because they use leaded aviation fuel. Jet and turbine aviation traffic do not use leaded fuel but are important to control for because they generate other pollutants, such as particulate matter, volatile organic compounds, and noise, which are associated with adverse cardiovascular morbidity and mortality [27]. Piston-engine aircraft in our sample were almost exclusively categorized as small equipment; jet aircraft were mostly larger sizes; and other turbine-engine aircraft were a mix of sizes. (Weight classes in the TFMSC database included heavy, B757, large jet, large commuter, medium commuter, and small equipment. We pooled the first five categories together and refer to these equipment types as “large.” For our study area and period, 99% of piston-engine operations, 1% of jet operations, and 34% of turbine-engine operations were categorized as small equipment).

Although the data were not as detailed, we were also interested in the number of general aviation VFR operations at each airport in each year. Because most general aviation activity uses piston-engine aircraft, and most other flight types do not [4], we used general aviation operations reported by ATADS and 5010 forms as a proxy for piston-engine VFR operations. We obtained the number of annual general aviation VFR flights from ATADS data when available. When ATADS data were unavailable, we used information from the FAA 5010 forms on general aviation operations. The 5010 forms did not distinguish between IFR and VFR general aviation operations, so we subtracted the number of piston-engine IFR operations indicated by TFMSC from the total number of general aviation operations reported by the 5010 forms to derive an estimate of general aviation VFR operations at these airports. Our data indicated that the number of general aviation IFR operations was highly correlated with the number of multi-engine piston aircraft based at airports, while the number of general aviation VFR operations was highly correlated with the number of single-engine piston aircraft based at airports. We excluded six airports from our analysis where TFMSC data were available but for which the general aviation VFR operations data were missing from the 5010 reports.

We linked each 2010 census block group in North Carolina to the closest TFMSC airport using geodesic distances calculated from each census block centroid to the nearest runway at the airport. A spatially explicit FAA data layer of the runway footprints was obtained from ESRI ArcGIS Online [38]. We conducted all GIS analyses using ArcMap 10.8.1. The distances for each block were then aggregated to the block group level by taking a population-based weighted average. We also recorded the number of runways at each airport.

**Figure 1:**
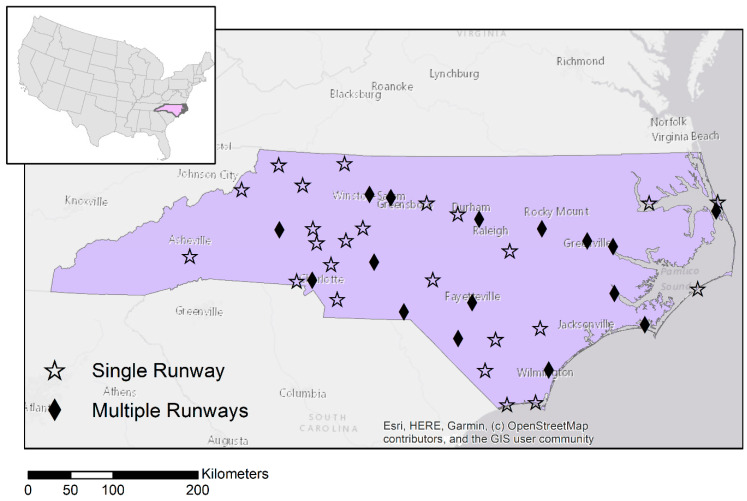
North Carolina airports with flight operations data available and populations located within 2 km.

**Figure 2:**
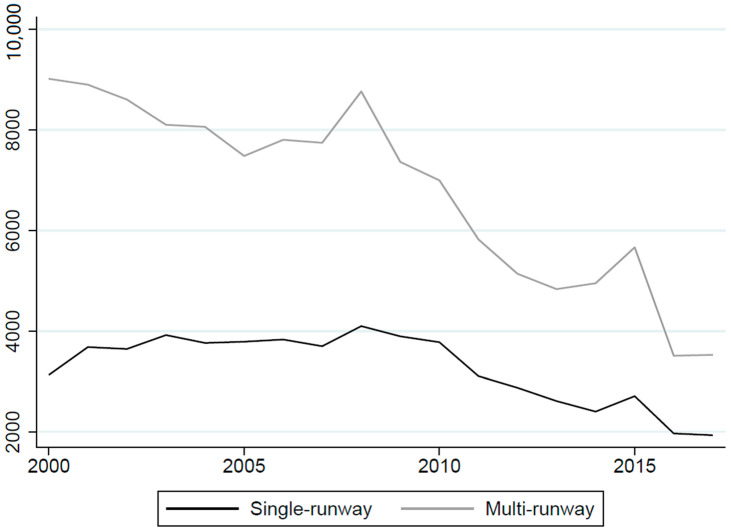
Annual average piston-engine IFR operations at single-runway and multi-runway airports in North Carolina, 2000–2017. Note: Annual averages were calculated using CEM weights assuming a 2 km treatment group (explained in Section 5).

We focused our analysis on forty North Carolina airports that met the following three criteria: (1) data on piston-engine IFR flight operations from TFMSC were available; (2) data on general aviation operations from ATADS or 5010 reports were available; and (3) there was at least one census block group with a centroid located within 2 km of the airport runway(s). We focused on airports with populations within 2 km because past empirical research found effects on children’s BLL to be concentrated within a few kilometers of airports [6,7]. Twenty-four of the forty airports included in the analysis had a single runway, and 16 had between two and four runways (Figure 1). Figure 2 shows the trend in annual average piston-engine IFR operations at single-runway and multi-runway airports during the study period.

In 2010, out of 6155 census block groups in North Carolina, 1764 fell within 10 km of one of the 40 airports shown in Figure 1. We focused our analysis on these block groups, which included 92,908, cardiovascular deaths among individuals 65 and over during the study period. While we hypothesized that any health effects from aviation fuel emissions are likely to be concentrated within a few kilometers of airport runways based on past literature [6,7], we included more distant block groups out to 10 km in the analysis as a less-exposed “control” group. No census block groups in our study were within 4 km of more than one TFMSC airport, though 1% of block groups were within 10 km of more than one TFMSC airport.

We incorporated demographic and socioeconomic variables for each block group into the analysis using data from the 2000 and 2010 Decennial Censuses of Population and Housing and the American Community Survey (ACS) five-year estimates for each five-year period from 2006–2010 to 2015–2019 downloaded from the IPUMS National Historical GIS Information System [39]. (We treated each set of ACS five-year estimates as representative of the midpoint year. For example, we considered 2006–2010 estimates as representative of the year 2008, 2007–2011 estimates as representative of 2009, and so on). These variables included the total population age 65 and older, the share of the population that was Black, the share of the population that was Hispanic or Latino, the share of the housing stock that was vacant, the share of occupied housing that was renter-occupied, the median household income, the share of the population age 25 and over with a college degree, and the share of housing stock built before 1950. Pre-1950 housing stock is another potential source of lead exposure due to the widespread use of leaded paint and plumbing in older homes. (While the federal bans on residential lead paint and lead service lines did not go into effect until 1978 and 1986, respectively, these sources are more likely to be present in pre-1950 houses [40,41]). We also used data on the total population and the area of each block group to calculate the population density. We linearly interpolated these variables from 2000 to 2008 to obtain estimates for each year in our study for which we did not have single-year or five-year estimates. (IPUMS NHGIS provides integrated census data over time for several variables, allowing us to include 2000 census block group data in our analysis based on 2010 block group identifiers for all census variables in our analysis except for median household income, percent of the adult population with a college degree, and the share of housing stock built pre-1950. For these three variables, we imputed values for the years 2000–2007 using the predicted values from regressing each variable on the 2010 value of the variable, year, and the other census variables in our analysis for 2008–2017). 

We controlled for exposure to stationary industrial sources of lead and other air toxics using data from EPA’s Risk-Screening Environmental Indicators (RSEI) model [42]. For each block group and year in our analysis, the RSEI geographic microdata provided lead air concentrations and aggregate toxicity-weighted concentrations of other pollutants attributable to emissions from stationary industrial sources that report to the Toxics Release Inventory. (Because TRI reporting requirements changed for lead and several other chemicals in 2001, we did not use RSEI data for the year 2000 and instead made the simplifying assumption that concentrations in 2000 were equal to concentrations in 2001. The measure of aggregate toxicity-weighted air concentrations only included chemicals whose reporting requirements have not changed since 2001). Airport emissions are not included in the Toxics Release Inventory.

We linked each census block group to non-TFMSC airports and heliports and roadways, which could also be sources of lead and other pollutants. Non-TFMSC airport and heliport locations were represented by FAA Airport Reference Points. Roadway data came from the US Census Bureau’s TIGER/Line files [43]. We also calculated the distance to the nearest hospital, since access to medical care can affect whether mortality occurs after a myocardial infarction or other life-threatening emergency [44,45]. The locations of hospitals in North Carolina were obtained from NCOneMap [46]. As with our measure of distances to airports, we calculated distances for each census block and then aggregated up to the block group level by taking a population-based weighted average. We also constructed a measure of the percent of each block group exposed to over 55 decibels of transportation noise from roadways and aviation using the 2016 National Transportation Noise Map [47]. The 2016 National Transportation Noise Map provided modelled estimates of aviation noise at 18 of the 40 airports in our analysis, representing 69% of block groups in the study area. We lacked temporal variation in these variables, so their effects were not identified in our primary models that included block group intercepts, but we included them in a spatially coarser airport intercept model.

We included two additional county-level control variables that could affect cardiovascular mortality trends over time: the unemployment rate and exposure to heat waves, as measured by the number of days exceeding 90 degrees Fahrenheit [48,49]. We obtained annual unemployment rate data from the U.S. Bureau of Labor Statistics’ Local Area Unemployment Statistics program [50]. We used data on daily temperatures from the National Oceanic and Atmospheric Administration’s Climate Data Search to construct our measure of days exceeding 90 degrees [51]. (Temperature data were missing for seven counties in North Carolina. We imputed values for these counties by taking the mean across counties in the same climatic region in North Carolina [52].

Similar to other studies of aircraft emissions (e.g., [7,21,28]), we incorporated wind direction into the analysis. We obtained data on wind direction during 2000 to 2017 from Iowa State University’s Iowa Environmental Mesonet [53]. The wind direction data were available for the entire study period at 27 of the 40 airports in our study and were available for part of the study period at seven more airports. Overall, wind direction data were available for 90% of block group-year observations in our analysis dataset. Using data on airport wind direction and the near angle of each block group in relation to the closest TFMSC airport runway, we calculated variables denoting the percent of days during each year of the study period during which a block group was downwind and upwind of the closest airport runway. An examination of these data showed a bimodal pattern of prevailing winds at many North Carolina airports, with winds blowing from the southwest for part of the year and then from the northeast for the other part of the year. We constructed wind variables for each airport for each year of the study period using all available wind direction observations of at least 5 miles per hour during daytime and evening hours during which flights typically occur (7 am to 10 pm). We calculated the percent of days in each year the wind was blowing from each of eight directions defined by dividing the compass rose into octants. We then calculated the percentages of days in each year during which a block group was located upwind or downwind of the airport using the near angle of the block group in relation to the airport. 

## 5. Empirical Analysis

Our outcome variable was the number of cardiovascular deaths among individuals age 65 and older in each block group and year. Our key exposure variables were proximity to the closest TFMSC airport and the numbers of piston-engine and general aviation flight operations at the closest TFMSC airport during the corresponding year.

We used a Poisson model to estimate the relationship between mortality and exposure to aircraft operations, adjusting for several other explanatory variables (see Table 2). The Poisson regression model and the closely related negative binomial regression model have been used in previous studies of environmental risk factors and disease incidence (e.g., [54,55]). The Poisson model is appropriate for count data censored at zero and yields consistent estimates when used with fixed effects [56,57]. While the negative binomial regression model is sometimes used as an alternative to the Poisson regression model for modeling over-dispersed count data, it can yield inconsistent parameter estimates when used with panel count data [57,58]. Seventeen percent of the block group-year observations in our study had zero cardiovascular deaths among individuals 65 and older. Ordinary Least Squares is not appropriate for count data because it can predict negative and non-integer values. We used the natural log of the total population age 65 and older in each block group in each year as the offset variable. The inclusion of the population offset allows us to interpret our model as estimating the cardiovascular mortality rate among the population of interest.

We first estimated a model to examine the association between cardiovascular mortality and proximity to the closest TFMSC airport. The model includes several control variables to adjust for other risk factors besides leaded aviation fuel emissions. These include the sociodemographic variables discussed above, industrial stationary source emissions, and, for 18 airports in our sample, a time-invariant measure of airport noise. While block group fixed effects would further control for time-invariant socioeconomic and geographic determinants of mortality, we did not include them in this model because they are perfectly collinear with the airport proximity variables, which are of primary interest. Because we did not use block group fixed effects, we interpreted the coefficients of this model as correlations rather than causal effects.

The airport proximity model can be written as:

(1) $$\;m_{icat}=exp\left(\ln\left(pop65_{it}\right)+\alpha_{1}D_{ia}+X_{ict}\beta +Y_{t}+B_{\alpha }+\tau_{\alpha }t\right)$$

Here, *m_icat_* represents the number of cardiovascular deaths in the 65 and older population in block group *i*, in county *c*, located closest to TFMSC airport *a*, during year *t*. We modeled *m_icat_* as a function of several variables, including the offset term representing the natural log of the number of people age 65 and older in block group *i* in year *t* (*pop*65*_it_*). The coefficient on the offset term was constrained to equal one. *D_ia_* is an indicator variable denoting that the population-weighted centroid of block group *i* is within a given distance of airport *a*. Due to the uncertainty about the spatial extent of any adverse effects from aircraft operations on cardiovascular mortality, we estimated the model three separate times using different distances to reflect possible treatment groups: 0–1, 0–2, and 0–3 km. To further examine heterogeneity with respect to distance, we also estimated a single regression that includes three mutually exclusive distance indicators: 0–1, 1–2, and 2–3 km. (In this model, *D_ia_* in Equation (1) is a vector of mutually exclusive indicators denoting incremental distance bins).

We included a vector of block group and county control variables (*X_ict_*), including airport noise, proximity to major roads, unemployment, and time-varying block group socioeconomic characteristics. Year-specific intercepts for each year of the analysis (*Y_t_*) were included to capture statewide trends over time. Separate intercepts denoting the closest TFMSC airport (*B_a_*) captured time-invariant location- and airport-specific factors affecting mortality rates, albeit at a relatively coarse geographic resolution. We also included a linear time trend that is specific to each airport, represented by $\tau_{\alpha }t$, to capture local trends over the study period. Coefficients to be estimated include $\alpha_{1}$, the correlation between airport proximity and mortality, and β, the effects of other characteristics on mortality. These coefficients represent the percent increase in cardiovascular mortality from a one-unit change in the corresponding explanatory variable.

To examine the effect of year-to-year changes in air traffic on cardiovascular mortality near airports, we turned to a different specification that exploits temporal variation in the number of piston-engine and other flight operations. This model includes block group-specific intercept terms $\left(B_{i\;}\right)$ to absorb time-invariant neighborhood characteristics affecting mortality at a much finer spatial resolution than the airport intercepts included in the airport proximity model. We did not include the airport noise or proximity variables in this specification because they are perfectly collinear with the block group intercepts. The higher resolution block group intercepts absorb at a more local scale all observed and unobserved time-invariant neighborhood characteristics that are correlated with cardiovascular mortality, including proximity to an airport (*D_ia_*) and characteristics of that airport ($B_{\alpha }$). We interacted airport proximity with different types of annual flight operations, including piston-engine IFR operations and other general aviation operations that typically use leaded fuel. Our inclusion of small and large jet- and turbine-engine flights that do not use leaded fuel helps to control for temporal variation in aviation noise and non-leaded fuel emissions that could have affected cardiovascular mortality.

The annual flight operations model can be written as:

(2) $$m_{\mathrm{icat}}=\exp(\mathrm{In}({\mathrm{pop}65}_{\mathrm{it}})+\gamma_{1}{\mathrm{PE}}_{\mathrm{iat}}+\gamma_{2}{\mathrm{LG}}_{\mathrm{iat}}+\gamma_{3}{\mathrm{SM}}_{\mathrm{iat}}+\gamma_{4}{\mathrm{GA}}_{\mathrm{iat}}+\delta_{1}D_{\mathrm{ia}}\;*{\mathrm{PE}}_{\mathrm{iat}}+\delta_{2}D_{\mathrm{ia}}*{\mathrm{LG}}_{\mathrm{iat}}+\delta_{3}D_{\mathrm{ia}}*{\mathrm{SM}}_{\mathrm{iat}}+\delta_{4}D_{\mathrm{ia}}*{\mathrm{GA}}_{\mathrm{iat}}+{\beta X}_{\mathrm{ict}}+Y_{t}+B_{i}+\tau_{\alpha }t)$$

We included the numbers of piston-engine (*PE_iat_*), large jet or turbine (*LG_iat_*), and small jet or turbine (*SM_iat_*) IFR aviation operations and the number of general aviation VFR operations (*GA_iat_*) at the closest airport *a* to block group *i* during year *t.* We interacted these aviation variables with *D_ia_*, the airport proximity indicator corresponding to block group *i*’s location. These interaction terms are our key explanatory variables representing possible exposure to leaded aviation fuel emissions near airports, given by D_ia_ * PE_iat_ and D_ia_ * GA_iat_. Therefore, $\delta_{1}$ and $\delta_{4}$ are the parameters of primary interest. They represent the percent change in cardiovascular mortality per piston-engine IFR operation and general aviation VFR operation at different distances from the airport. Like the airport proximity model, we estimated the flight operations model using three separate regressions with different distances in each regression (0–1, 0–2, and 0–3 km). We also estimated a regression including mutually exclusive distances (0–1, 1–2, and 2–3 km). 

We clustered the standard errors by the closest airport in the airport proximity model and by census tract in the annual flight operations model (Equations (1) and (2), respectively). We used census tracts instead of block groups as the unit for clustering in the annual flight operations model to allow for any unobserved spatial correlation in cardiovascular mortality that may occur within broader neighborhoods.

We separately estimated these models for block groups near single-runway and multi-runway airports. We disaggregated by the number of runways because we anticipated that our measure of proximity to airport traffic is more precise for single-runway airports. For airports with more than one runway, there may have been greater dispersion of emissions across space, and we lacked data to apportion flight operations to specific runways at each airport, creating uncertainty about where the emissions occurred and hence which block groups were more exposed to air traffic emissions. This potential for classical measurement error in our measure of air traffic exposure at multi-runway airports could bias our estimates of the impact of aircraft operations on mortality towards the null.

We also estimated specifications of the airport proximity and annual flight operations models that incorporated wind direction. For the airport proximity model, we interacted the percentages of days a block group was downwind and upwind of the closest TFMSC airport with the indicators for distance from the airport. For the annual flight operations model, we added terms capturing the three-way interactions between annual flight operations, distance from the airport, and percentages of each year the block group was downwind and upwind from the airports. In these models, we also included a dummy variable indicating block group-year observations for which wind direction data were unavailable (corresponding to 10 percent of observations).

Our original models that do not include interaction terms with wind direction estimate average effects for all populations equidistant around airport runways. These estimates do not reflect spatial heterogeneity that could arise if wind dispersion of lead particles causes higher lead exposures from piston-engine air traffic for populations downwind and lower exposures for those upwind of airports. However, because runways often run parallel with the prevalent wind direction, models assuming directional homogeneity (as in Equations (1) and (2)) could be more appropriate for reflecting exposures to populations living along the sides of runways, where startup, idling, and taxiing emissions could also generate high concentrations of lead in the air.

To ensure that the “treated” block groups located within *D_ia_* of a TFMSC airport are comparable in terms of socioeconomic characteristics that could affect cardiovascular mortality to the “control” block groups located farther away (but still within 10 km), our primary estimates used coarsened exact matching (CEM) [14]. CEM is a pre-processing algorithm that identifies observations in the treatment and control groups that match in terms of all explanatory variables selected by the analyst, after first coarsening the continuous variables into discrete categories. CEM also derives weights to balance the matched distributions of the observed socioeconomic characteristics across the treatment and control groups. All treatment observations that do not have an identical “match” in the control group (and vice versa) are assigned a weight of zero and dropped from the sample.

We matched our treated and control samples using seven coarsened block group sociodemographic variables and three non-coarsened variables. The coarsened variables include median income, population density, the share of the adult population that graduated from college, the share of the population that was Black, the share of population that was 65 and older, the share of housing that was renter-occupied, and the share of housing that was built before 1950. We divided median income into tertiles, and for all other coarsened variables we created three categories defined by equally spaced cut points. We matched on the 2010 values for all census variables. The three variables used for an exact match were county, year, and closest airport. In balancing observable sociodemographic characteristics across the treatment and control groups, this strategy may also increase similarity in unobservable traits that are correlated with the observable characteristics.

We generated three sets of CEM weights corresponding to three possible cutoffs delineating the treatment and control groups already discussed: 1 km, 2 km, and 3 km from the closest TFMSC airport. The Supplementary Materials also present regression results using the full, unweighted sample. While we found that the results were robust across the two approaches, the CEM estimates are preferred because they improved the balance in sociodemographic covariates across block groups closer versus farther from airports in our sample, as discussed in Section 6.

Our preferred approach combining panel data, block group-specific intercepts, and matching allows us to more credibly isolate the effect of piston-engine aviation on cardiovascular mortality based on year-to-year changes in air traffic. The airport proximity model (Equation (1)) is less able to isolate this effect because airport proximity could be correlated with socioeconomic or other local attributes affecting cardiovascular mortality. The flight operations model interacting distance from the airport with annual aviation operations (Equation (2)) is conceptually similar to a quasi-experimental difference-in-difference model, although the exposure variable representing number of flight operations varies continuously over time rather than changing discretely at one point in time.

## 6. Results

Our full sample included 31,495 census block group-year observations. (This sample excluded 357 block group-year observations estimated to have zero individuals 65 and older, 61 observations with zero housing units, and 1 observation with an estimated cardiovascular mortality rate greater than one). Using CEM to focus our analysis on a more homogenous matched sample of treated and control block groups greatly reduced the sample size. Using a treatment definition of 2 km, we kept 81% of treatment observations and 28% of control observations and were left with 9997 observations. Since most of the “pruned” observations were in the control group, we retained most observations in the treatment group closest to each TFMSC airport. Therefore, CEM helped us to identify the most appropriate counterfactual set of block groups. Using a treatment definition of 1 km yielded a much smaller matched sample of 1552 observations, while a treatment definition of 3 km yielded a larger matched sample of 14,245 observations.

Table 2 presents summary statistics for the CEM-weighted sample using the 2 km treatment definition. Table S2 in the Supplementary Materials presents summary statistics for all variables for the full sample, without matching. We present these statistics separately for single-runway and multi-runway airports. The matched treatment and control groups near single-runway airports have similarly high incomes and education levels, though the control group has somewhat higher rates of vacant, rental, and older housing; a higher percentage of Black residents; and a higher population density. Average air concentrations for lead and for aggregate toxicity-weighted emissions from stationary industrial sources were similar across the treatment and control groups, though the control group included more block groups located within 4 km of Charlotte Motor Speedway, a source of lead emissions before 2007.

The matched treatment and control groups near multi-runway airports have sociodemographic and housing characteristics that are similar to each other, though incomes were somewhat higher in the control group. Air lead concentrations from stationary industrial sources were also higher in the control group. The differences between the single-runway and multi-runway samples are more pronounced than the differences between the treatment and control groups for each airport type. Income and education levels were much higher, and the share of Black and Hispanic residents was much lower, near single-runway airports. These divergent demographic characteristics further support our decision to analyze single-runway and multi-runway airports separately.

**Figure 3:**
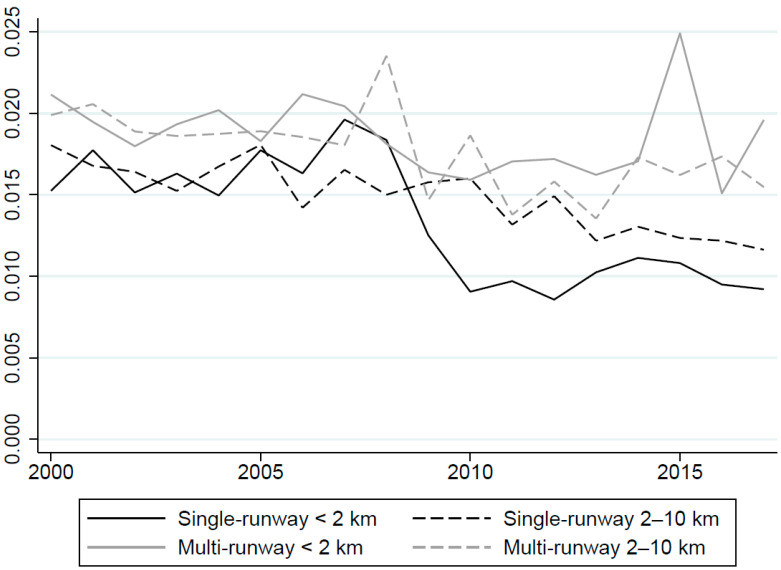
Cardiovascular mortality rate (age 65 and older) by airport type and distance from airport: CEM sample. Annual averages calculated using CEM weights assuming a 2 km treatment group.

Figure 3 shows cardiovascular mortality rates near single- and multi-runway airports during the study period in the matched and weighted sample, again using the 2 km cutoff to define treatment and control groups near each airport type. The figure shows that cardiovascular mortality rates were somewhat lower near single-runway airports than multi-runway airports. Though there was substantial year-to-year variation, mortality rates were generally similar across the treatment and control groups near each airport type. Figure S1 in the Supplementary Materials shows trends for the full unweighted sample, revealing a larger divergence between the treatment and control groups for each airport type than the matched sample. This outcome suggests that CEM helped to identify treatment and control groups that are similar in terms of the broad determinants of cardiovascular mortality during the study period.

Table 3a presents the airport proximity coefficients from the regressions corresponding to Equation (1) for the single- and multi-runway airport samples. We estimated three separate regressions, varying the cutoff between the treatment and control areas from 1 km to 3 km. The treatment definition used for deriving the CEM weights matches the distance variable included in each regression. We did not find a positive relationship between cardiovascular mortality and proximity to single-runway TFMSC airports. In fact, cardiovascular mortality rates were significantly lower within 1 km of single-runway airports, and there was no statistically significant association using the 0–2 km or 0–3 km treatment definitions. In contrast, cardiovascular mortality rates were higher closest to multi-runway airports. Mortality was 12 to 15 percent higher within 0–1 km and 0–2 km of multi-runway airports, though the estimate was only statistically significant using the 0–2 km treatment definition. There was no significant association between mortality and proximity to multi-runway airports beyond this distance.

Table 3b presents the airport proximity coefficient estimates from a single regression model that includes mutually exclusive distance bins in 1-km increments. This model used the CEM sample and weights derived using the 3 km treatment group so that all of the distance bins included in the regression fall within this treatment group. The results were similar to those shown in Table 3a. Cardiovascular mortality rates were lower near single-runway airports and higher near multi-runway airports, though the latter effect was not statistically significant in this model. Because the models in Table 3a,b do not include spatially refined block group intercepts, we cannot parse to what extent these associations are due to residual confounding with factors common to these neighborhoods or due to other lead- and non-lead-related hazards at these airports. (In the Supplementary Materials, Table S3 shows the full set of coefficient estimates for all control variables included in this regression. Table S4 shows the key coefficient estimates for this model using the full sample, without CEM weights).

Table 4a presents the key coefficients from the flight operations regressions (Equation (2)). This model is our preferred specification for estimating the unbiased effect of piston-engine air traffic on cardiovascular mortality. Similar to Table 3a, we present results from three different regressions in which the treatment definition (and corresponding CEM weights) varies from 1 to 3 km. The results indicate that piston-engine IFR flights have a statistically significant adverse effect on cardiovascular mortality for treatment groups that extend up to 3 km away from single-runway airports. The effect is largest within 1 km of the runway and declines monotonically as we expand the treatment group to include block groups farther from the nearest single-runway airport. The coefficient estimates indicate that each piston-engine flight operation at a single-runway airport increased cardiovascular mortality by 0.07 percent in the 0–1 km treatment group, 0.01 percent for the 0–2 km treatment group, and 0.007 percent for the 0–3 km treatment group.

We also found a significant increase in cardiovascular mortality of 0.03 percent per piston-engine IFR operation within 1 km of multi-runway airports but found no significant effect from changes in piston-engine IFR operations beyond this localized area. As already noted, at multi-runway airports, we have lower confidence that the distance between populations and airport runways is a good proxy for exposure to leaded fuel emissions because we do not know which runway was used for each operation.

Turning to the other flight types, the results suggest that general aviation VFR flights were negatively associated with cardiovascular mortality within 0–1 km of multi-runway airports but had no statistically significant effect on cardiovascular mortality at any other distances. The negative association is counterintuitive, but as already noted, our VFR flight data lack accurate year-to-year variation at most airports given their exclusion from the TFMSC database. In addition, while most general aviation VFR flights are thought to be piston-engine, our data did not confirm engine type (and, hence, the use of leaded fuel). Moreover, piston-engine IFR operations are more likely than VFR operations to be performed by twin engine aircraft, which have higher lead emissions per operation than single-engine aircraft.

The effects from small and large jet or turbine flights on cardiovascular mortality are also not significantly different from zero in most specifications. The exception is a significant adverse effect of large jet and turbine operations within 0–1 km of single-runway airports and a marginally significant adverse effect of small jet and turbine operations on cardiovascular mortality within 0–3 km of single-runway airports. Large jet and turbine aircraft have the potential to cause adverse effects from both conventional pollutant emissions and noise near airports. In addition, if smaller jet and turbine operations emit very fine particulates that disburse with wind, then adverse health effects could occur further from airports. The magnitude of the coefficients on small and large jet/turbine operations at single-runway airports is similar to that of the piston-engine IFR coefficients using the 0–3 km treatment definitions. Overall, these results suggest that piston-engine IFR flights were relatively more harmful to cardiovascular health than similarly sized flights in areas located closest to single-runway airports but that these effects may converge or even reverse a few kilometers away from the airports.

Table 4b presents results of the flight operations model using a single regression that interacts flight operations with mutually exclusive distance bins of 0–1, 1–2, and 2–3 km from the closest airport. Consistent with the results in Table 4a, we found statistically significant effects of piston-engine operations in block groups with centroids 0–1, 1–2, and 2–3 km from single-runway airports. Each piston-engine IFR flight operation increased cardiovascular mortality by 0.09 percent, 0.02 percent, and 0.005 percent at these distances, respectively.

In contrast to Table 4a, the estimated effect of piston-engine IFR operations within 1 km of multi-runway airports was smaller and not statistically different from zero. The main difference between these models is the treatment and control group definitions used to calculate the CEM sample and weights. The small number of block group-year observations within 1 km of the closest airport contributed to our uncertainty about the effect of air traffic operations near multi-runway airports.

The estimates of the effects of other flight types on cardiovascular mortality from Table 4b are generally similar to those from Table 4a, except that the adverse effect of large jet and turbine operations within 0–1 km of single-runway airports is not statistically significant.

Table S5 in the Supplementary Materials presents the full set of coefficients for all explanatory variables for the CEM-weighted flight operations model corresponding to Table 4b. These results suggest that annual increases in cardiovascular mortality were significantly associated with increases in the share of rental housing and the adult population that graduated from college, decreased population density, increases in toxicity-weighted air pollutant concentrations, and more days exceeding 90 degrees in communities near single- and/or multi-runway airports. We also observed lower cardiovascular mortality rates within 4 km of Charlotte Motor Speedway prior to the voluntary phaseout of lead in racing fuel. While this result is counterintuitive, we note that there are only four census block groups within 4 km of the Speedway in the matched sample, so the sample size on which this comparison is based is extremely small.

The primary finding that piston-engine IFR operations had a statistically significant adverse association with cardiovascular mortality within 0–1 and 1–2 km from single-runway airports is robust to alternative specifications that use the full sample without CEM weights and to models that exclude other operation types Table S6 in the Supplementary Materials presents regression results using the full sample without CEM weights. These results are extremely similar to the CEM-weighted model results shown in Table 4b. As shown in Table S7 in the Supplementary Materials, the piston-engine IFR operation coefficient estimates are similar to those in Table 4b when all other operation types except for piston-engine IFR traffic are excluded. This suggests that the primary results are not being driven by collinearity with the other flight operation variables.

We provide an illustrative example of the magnitude of the adverse effect of piston-engine aircraft on cardiovascular mortality by calculating the impact of a 10 percent reduction in operations near single-runway airports. Using the coefficients for the 0–2 km treatment effect from column 1 of Table 4a, we estimated that reducing piston-engine IFR operations at single-runway airports by 10 percent, which is equivalent to 258 takeoffs or landings per airport on average, would result in a statistically significant 3 percent reduction in annual cardiovascular mortality among individuals age 65 and older, assuming that all other flight traffic is held constant. This equates to a drop in cardiovascular deaths of 0.085 per block group, which totals to approximately five avoided deaths per year across all block groups located within 2 km of one of the 24 single-runway airports in the study area. If we instead assume that the reduction in piston-engine IFR flights is balanced by an equal increase in the number of small jet or turbine IFR operations (which do not use leaded fuel), we obtained a net reduction in cardiovascular mortality of 2 percent, equivalent to approximately three deaths per year across all block groups located within 2 km of a single-runway airport in the study area. (This net effect when assuming an offsetting increase in small jet or turbine IFR operations is not significantly different from zero (*p* = 0.28)). The latter illustration is of interest because it may better isolate the effects associated with leaded fuel emissions. Small jet and turbine IFR flights do not use leaded fuel but may emit similar levels of other pollutants and noise that could contribute to cardiovascular mortality.

Next, we consider how wind direction modifies the relationship between exposure to piston-engine aircraft traffic and cardiovascular mortality. Table 5 presents the key coefficients from a model similar to that presented in Table 3b, except that the 1-km incremental airport proximity bins were also interacted with the percentages of time the block group was downwind and upwind of the closest airport runway. We found that cardiovascular mortality rates were higher downwind within a few kilometers of both single- and multi-runway airports. Near single-runway airports, cardiovascular mortality was highest downwind within 1 km of the runway, and the downwind proximity coefficients decreased monotonically out to 3 km. Cardiovascular mortality rates were also lower upwind within each proximity bin out to 3 km from the runway. For the most part, the wind interaction coefficients were not statistically significant when considered individually. Jointly, the six proximity-wind coefficients were marginally significant (*p* = 0.08). The wind interaction model suggests that the counterintuitive negative association seen within 1 km of single-runway airports in Table 3a,b was concentrated upwind of runways and did not extend downwind.

Near multi-runway airports, cardiovascular mortality rates were significantly higher downwind within 1 km of the closest runway. The other proximity-wind coefficients in the multi-runway model did not show a clear monotonic trend and were not statistically significant. Overall, these results suggest that cardiovascular mortality rates among individuals 65 and over were higher near and downwind (but not upwind) of both single- and multi-runway airports. However, they do not identify to what extent these effects were caused by aircraft operations or by residual confounding from other cardiovascular disease risk factors that could be correlated with living downwind of airports.

Table 6 presents the coefficients from interacting runway proximity, wind direction, and annual flight operations. Similar to Table 4b, we interacted the three 1-km incremental proximity bins with annual piston-engine IFR aircraft operations, but we also included three-way interaction terms between airport proximity, piston-engine IFR operations, and location downwind and upwind of the nearest runway. We focused on piston-engine IFR operations in this model and excluded the other aircraft types due to concerns about multicollinearity from including so many terms multiplied by the same upwind and downwind variables. (Table S7 in the Supplementary Materials shows that in the primary model without wind direction interactions, the piston-engine IFR operation coefficients do not change substantively when other flight operations are excluded).

Similar to Table 4a, the results still show higher cardiovascular morality rates within 0–1 and 1–2 km of single-runway airports during years with more piston-engine IFR operations in general. In addition, within the 0–1 km distance bin, cardiovascular mortality rates were significantly higher downwind and significantly lower upwind of the runway. They were also significantly lower upwind at distances beyond 3 km from the closest runway. Adverse cardiovascular effects within 1–2 and 2–3 km of the runway were not significantly modified by location upwind or downwind. We note that it is possible for piston-engine aircraft to emit lead along the entire runway length during idling, taxiing, and run-up.

Near multi-runway airports, cardiovascular mortality rates were not significantly higher closer to the runways in years with more piston-engine IFR operations closer. The downwind and upwind interaction terms also lack statistical significance both individually and jointly (*p* = 0.12 for test of joint significance). These results are consistent with our previous findings of no detectable adverse effects from year-to-year changes in piston-engine operations near these larger, multi-runway airports.

## 7. Discussion

Our analysis indicates that increases in annual IFR piston-engine air traffic are associated with significant increases in cardiovascular mortality among adults age 65 and older living near single-runway airports. However, our data have several limitations—most notably, the lack of data on aviation lead air emissions, which are the exposures of interest. Blood lead surveillance data for adults or monitored air lead concentration data would provide a more precise measure of lead exposure from piston-engine air traffic. Our reliance on airport proximity and aircraft operations as imprecise proxies for exposure to lead emissions could lead to bias in our estimates. This bias could be downward due to classical measurement error or upward due to the confounding of lead emissions with other air emissions and noise from piston-engine operations.

Our measure of general aviation VFR flights is particularly coarse as an indicator of lead exposure. Our data source for VFR general aviation flights did not distinguish between piston- and non-piston-engine aircraft, though EPA has noted that most of these flights are piston-engine [4]. VFR flight data are not updated annually for most airports in our sample, introducing error into the temporal variation that we rely on for identification in our preferred regression models. These limitations could contribute to our lack of a significant association between general aviation VFR flights and cardiovascular mortality in most models, as well as a counterintuitive negative association in some models. VFR flights are more numerous than piston-engine IFR flights, so they remain a potentially important source of lead emissions.

Because leaded fuel usage can vary across parts of an airport, there is also uncertainty about the specific location of the lead emissions, leading to classical measurement error. This source of measurement error should be less pronounced for single-runway airports. Our use of population-weighted block group centroids instead of individual residential addresses to calculate both distance and downwind/upwind measurements exacerbates this source of measurement error, further biasing our results toward the null. However, our finding of more pronounced adverse effects near single-runway airports is consistent with other research finding higher soil lead levels near single-runway airports in Oklahoma [22].

We incorporated wind direction to account for potential spatial heterogeneity in air lead exposure at different distances to runways. However, our approach was not a spatially or temporally refined atmospheric dispersion model that would reflect variation in factors such as meteorological conditions, topography, and particulate sizes. Coupled with our proximity and aircraft operations variables, our measures of the percent of time each population-weighted block group centroid was downwind or upwind of the nearest runway provide a relatively coarse proxy for air lead exposure.

Our primary analysis focused on the effects of year-to-year fluctuations in piston-engine air traffic. A key uncertainty in the scientific literature is the timing and duration of lead exposure resulting in adverse cardiovascular effects [1]. If cardiovascular damage accrues over many years of exposure, which is likely the case, our results underestimate the total contribution of piston-engine air traffic to cardiovascular mortality.

Our study was also limited to airports large enough to report data on piston-engine IFR flights to FAA, as reflected in the TFMSC database. We cannot assume that estimates of the impact of aviation traffic at larger airports are generalizable to smaller airports, which are likely to have fewer based aircraft and flight operations. Some of these smaller airports could be located closer to residential neighborhoods than larger airports if local zoning does not require setbacks, so lead emissions from these airports could still raise public health concerns. We did not conduct an analysis of proximity to smaller airports as a proxy for exposure because airport location could be correlated with socioeconomic and other determinants of cardiovascular mortality, making it challenging to isolate the effect of piston-engine air traffic holding all other risk factors constant with this approach.

As already noted, there is potential for omitted variable bias if neighborhoods near airports were systematically different from those farther away. We used several strategies to minimize this potential bias, particularly in the flight operations regressions. These strategies included spatially refined census block group intercepts, coarsened exact matching on several socioeconomic characteristics that were associated with cardiovascular mortality rates, and the inclusion of numerous time-variant control variables and linear airport-specific time trends. To minimize bias due to the potential confounding of aircraft lead emissions with aircraft noise and other pollutants that can cause cardiovascular damage, we adjusted for aviation noise in our airport proximity regressions and accounted for large and small jet- and turbine-engine operations in our flight operations models. Despite this multi-pronged approach, we cannot assert with 100% confidence that we eliminated all sources of bias.

Given the potential for our results to be biased towards the null, it is notable that we estimated statistically significant increases in annual cardiovascular mortality from an increase in piston-engine IFR air traffic for block groups up to 2 or 3 km of single-runway airports. The magnitude of these effects is similar to findings from other studies examining associations between airport pollution and cardiovascular disease. For example, Schlenker and Walker found that a one standard deviation increase in aircraft carbon monoxide emissions caused a 9 percent increase in daily mean hospital admissions for heart problems near California airports [28]. This result corresponds to a 1.4 percent increase in hospital admissions per 10 percent increase in carbon monoxide. Correia et al. found that hospitalization for cardiovascular disease was 3.5% higher in zip codes with 10 dB higher 90th centile aviation noise exposure [25]. This finding roughly translates to a 1.8 percent increase in cardiovascular disease per 10 percent increase in 90th centile aviation noise. However, our findings are only applicable to a highly localized area near single-runway airports, whereas these studies found effects surrounding broader geographic areas near major airports.

## 8. Conclusions

Piston-engine aviation is the largest remaining source of airborne lead emissions in the United States. Our study is the first to estimate the effect of piston-engine aircraft operations on cardiovascular mortality among adults age 65 and older. The findings are mixed but suggestive of adverse effects. We found higher cardiovascular mortality rates within a few kilometers downwind of airport runways, though these results were not always statistically significant. We did not consistently find that cardiovascular morality was significantly higher in years with more piston-engine IFR operations at multi-runway airports, nor do we find higher mortality in years with more general aviation VFR operations, possibly because our measure of leaded avgas exposure is less precise in these cases. Spatial precision is important in contexts like this one, where the disamenity is very localized. However, we found that adults age 65 and older living within a few kilometers and downwind of single-runway airports had significantly higher cardiovascular mortality rates in years with more piston-engine IFR operations compared to adults living farther away from these airports.

To obtain more reliable and precise estimates of the effect of leaded aviation fuel emissions on cardiovascular outcomes, a direction for future research is to conduct similar analyses using a larger sample of airports with reliable flight operations data. Data on the variation in air lead concentrations and/or adult blood lead levels over time near airports would further expand opportunities to identify the health effects of changes in adult lead exposure. In addition, a more comprehensive dataset of individual-level health and location data for the entire population of adults age 65 and older living near airports—not just those who experience a fatality or other significant adverse effect—would allow for a more spatially refined analysis based on the distance from individuals’ residences to the closest airport runway. In the meantime, our study presents preliminary evidence that reducing emissions from leaded aviation fuel could have significant health benefits for adult populations who are often overlooked in discussions of lead exposure.

## Figures and Tables

**Table 1:** FAA data sources for general aviation and/or piston-engine operations.

Data Source	Number of North Carolina Airports Included	Engine-Type Information Available?	Includes Instrument Flight Rules (IFR) and Visual Flight Rules (VFR) Operations?	Reporting Frequency	Earliest Date Available
5010 forms	>400 (but only 178 had non-missing operations data)	No	Yes (but not reported separately)	Annually for some airports, but often less frequent	1998
Traffic Flow Management System Counts (TFMSC)	72	Yes	No (IFR only)	Daily	2000
Air Traffic Activity Data System (ATADS)	11	No	Yes	Daily	1990

**Table 2:** Summary statistics by airport type and distance from airport: CEM sample.

	Single-Runway Airports		*p*-Value of Difference in Means	Multi-Runway Airports		*p*-Value of Difference in Means
	0–2 km	2–10 km		0–2 km	2–10 km	
		Outcome variable				
65+ CVD mortality rate	0.014	0.015	0.050	0.019	0.018	0.17
	(0.016)	(0.015)		(0.018)	(0.019)	
		Exposure variables				
Piston-engine IFR operations	2585.00	2593.31	0.94	4485.21	4655.30	0.23
	(2913.69)	(2967.75)		(3948.67)	(4054.68)	
Large jet/turbine IFR operations	1804.38	1767.71	0.85	41,810.93	53,614.51	0.01
	(4867.35)	(4860.38)		(116,254.7)	(134,799.3)	
Small jet/turbine operations	784.55	764.31	0.68	2450.56	2592.02	0.08
	(1242.28)	(1255.12)		(2227.73)	(2338.34)	
General aviation VFR operations	26,709.10	26,691.02	0.98	24,498.62	24,140.24	0.39
	(18,421.99)	(18,418.29)		(11,889.09)	(11,793.64)	
		Time-variant control variables				
65+ population	201.69	220.16	<0.01	170.79	179.69	0.04
	(139.96)	(184.57)		(111.33)	(121.73)	
Share Black population	0.08	0.10	<0.01	0.41	0.37	<0.01
	(0.1)	(0.14)		(0.31)	(0.3)	
Share Hispanic population	0.06	0.06	0.12	0.08	0.08	0.91
	(0.07)	(0.07)		(0.12)	(0.1)	
Population density	0.0004	0.0005	<0.01	0.0007	0.0007	0.01
	(0.0005)	(0.0006)		(0.0006)	(0.0007)	
Percent vacant housing	0.17	0.19	<0.01	0.12	0.14	<0.01
	(0.19)	(0.22)		(0.08)	(0.14)	
Percent rental housing	0.27	0.29	0.05	0.50	0.48	0.03
	(0.19)	(0.21)		(0.24)	(0.25)	
Median income (2010, USD)	53,311.27	53,870.52	0.56	32,361.02	34,756.85	<0.01
	(19,716.4)	(25,083.26)		(15,159.38)	(17,513.37)	
Percent of adults 25+ with college degree	0.33	0.33	0.74	0.17	0.18	0.11
	(0.2)	(0.21)		(0.14)	(0.14)	
Percent pre-1950 housing	0.05	0.08	<0.01	0.15	0.14	0.04
	(0.05)	(0.07)		(0.13)	(0.14)	
Days above 90 degrees	33.74	33.89	0.88	41.49	41.41	0.92
	(25.08)	(24.64)		(22.57)	(22.95)	
Unemployment rate	6.40	6.39	0.95	6.84	6.85	0.9
	(2.54)	(2.54)		(2.56)	(2.55)	
Toxicity-weighted lead air concentration	2.12	2.04	0.58	4.20	8.42	<0.01
	(3.2)	(3.5)		(7.87)	(31.64)	
Toxicity-weighted total air concentration of chemical releases	6563.45	7402.34	0.65	21,018.46	26,613.49	0.24
	(38,800.27)	(47,412.59)		(80,941.03)	(145,346.2)	
Charlotte Motor Speedway located within 4 km × pre-2007 lead phaseout	0.01	0.00	<0.01	0.00	0.00	-
	(0.09)	(0.05)		(0)	(0)	
Percent days downwind	0.05	0.05	0.17	0.08	0.08	0.20
	(0.03)	(0.03)		(0.03)	(0.03)	
Percent days upwind	0.05	0.05	<0.01	0.07	0.07	0.39
	(0.03)	(0.04)		(0.03)	(0.04)	
	Time-invariant variables (only included in airport intercepts models)					
Percent > 55 decibel transportation noise	4.70	3.09	<0.01	10.01	5.00	<0.01
	(5.41)	(2.48)		(11.58)	(5.82)	
Heliport located within 2 km	0.03	0.11	<0.01	0.06	0.12	<0.01
	(0.18)	(0.31)		(0.23)	(0.32)	
Major road located within 2 km	0.95	0.86	<0.01	0.96	0.96	0.38
	(0.21)	(0.35)		(0.2)	(0.19)	
Major road located within 500 m	0.15	0.30	<0.01	0.36	0.39	0.04
	(0.35)	(0.46)		(0.48)	(0.49)	
Hospital located within 2 km	0.04	0.12	<0.01	0.10	0.18	<0.01
	(0.2)	(0.32)		(0.3)	(0.38)	
N	774	3607		980	4526	

Means calculated using CEM weights using 2 km treatment group. Standard deviations in parentheses. Percent downwind days and percent upwind days data only available for 90% of the sample. Percent > 55 decibel transportation noise data only available for 69% of the sample.

**Table 3:** (**a**) Key coefficient results from separate regressions with varying treatment cutoffs: Association between proximity to TFMSC airports with age 65+ cardiovascular mortality. (**b**) Key coefficient results from single regression with 3 km treatment cutoff: Association between proximity to TFMSC airports with age 65+ cardiovascular mortality.

(a)		
	Single-Runway Airports	Multi-Runway Airports
0–1 km	−0.288 **	0.120
	(0.143)	(0.0973)
Observations	396	1156
Pseudo R2	0.109	0.0977
0–2 km	−0.0505	0.154 ***
	(0.0816)	(0.0557)
Observations	4381	5506
Pseudo R2	0.114	0.0602
0–3 km	0.0124	0.0579
	(0.0467)	(0.0529)
Observations	6365	7880
Pseudo R2	0.104	0.0681
**(b)**		
	**Single-Runway Airports**	**Multi-Runway Airports**
0–1 km	−0.225 **	0.104
	(0.0942)	(0.128)
1–2 km	−0.00456	0.108
	(0.0719)	(0.0752)
2–3 km	0.0359	0.0132
	(0.0733)	(0.0706)
Observations	6365	7880
Pseudo R2	0.104	0.0684

(**a**) All models use CEM weights (calculated using a treatment definition consistent with the treatment group for each regression) and include closest TFMSC airport fixed effects, year fixed effects, airport-year time trends, and time-variant and time-invariant control variables shown in Table 2. Robust standard errors clustered by closest airport are in parentheses. *** *p* < 0.01, ** *p* < 0.05, * *p* < 0.1. (**b**) This model uses CEM weights (calculated using the 3 km treatment group) and includes closest TFMSC airport fixed effects, year fixed effects, airport-year time trends, and time-variant and time-invariant control variables shown in Table 2. Robust standard errors clustered by closest airport are in parentheses. *** *p* < 0.01, ** *p* < 0.05, * *p* < 0.1.

**Table 4:** (**a**) Key coefficient results from separate regressions with varying treatment cutoffs: Effect of annual flight operations on cardiovascular mortality near TFMSC airports. (**b**) Key coefficient results from single regression with 3 km treatment cutoff: Effect of annual flight operations on cardiovascular mortality near TFMSC airports.

(a)		
	Single-Runway Airports	Multi-Runway Airports
Piston-engine IFR operations * 0–1 km	0.000721 *	0.000323 ***
	(0.000382)	(0.000114)
Large jet/turbine IFR operations * 0–1 km	0.00116 ***	5.02 × 10^−5^
	(0.000275)	(0.000104)
Small jet/turbine IFR operations * 0–1 km	0.000164	9.84 × 10^−5^
	(0.00112)	(0.000127)
General aviation VFR operations * 0–1 km	−1.33 × 10^−5^	−2.81 × 10^−5^ ***
	(9.06 × 10^−6^)	(9.00 × 10^−6^)
Observations	396	1156
Pseudo R2	0.185	0.155
Piston-engine IFR operations * 0–2 km	0.000125 **	1.04 × 10^−5^
	(4.95 × 10^−5^)	(2.56 × 10^−5^)
Large jet/turbine IFR operations * 0–2 km	−2.21 × 10^−5^	−8.56 × 10^−7^
	(3.79 × 10^−5^)	(3.02 × 10^−6^)
Small jet/turbine IFR operations * 0–2 km	3.90 × 10^−5^	4.39 × 10^−5^
	(9.21 × 10^−5^)	(5.39 × 10^−5^)
General aviation VFR operations * 0–2 km	1.83 × 10^−6^	−3.60 × 10^−6^
	(2.78 × 10^−6^)	(5.26 × 10^−6^)
Observations	4381	5506
Pseudo R2	0.193	0.129
Piston-engine IFR operations * 0–3 km	7.91 × 10^−5^ ***	−9.40 × 10^−6^
	(2.38 × 10^−5^)	(1.94 × 10^−5^)
Large jet/turbine IFR operations * 0–3 km	3.30 × 10^−5^	1.99 × 10^−6^
	(5.23 × 10^−5^)	(1.68 × 10^−6^)
Small jet/turbine IFR operations * 0–3 km	8.84 × 10^−5^ *	4.78 × 10^−5^
	(5.34 × 10^−5^)	(4.68 × 10^−5^)
General aviation VFR operations * 0–3 km	−2.54 × 10^−6^	1.35 × 10^−6^
	(2.36 × 10^−6^)	(4.03 × 10^−6^)
Observations	6365	7880
Pseudo R2	0.189	0.145
**(b)**		
	**Single-Runway Airports**	**Multi-Runway Airports**
Piston-engine IFR operations * 0–1 km	0.000857 **	1.26 × 10^−5^
	(0.000361)	(5.78 × 10^−5^)
Large jet/turbine IFR operations * 0–1 km	0.000282	5.50 × 10^−5^
	(0.000195)	(0.000102)
Small jet/turbine IFR operations * 0–1 km	−4.06 × 10^−5^	4.99 × 10^−5^
	(0.00110)	(0.000143)
General aviation VFR operations * 0–1 km	−2.66 × 10^−6^	−1.10 × 10^−5^ ***
	(4.47 × 10^−6^)	(3.99 × 10^−6^)
Piston-engine IFR operations * 1–2 km	0.000150 ***	1.22 × 10^−5^
	(4.88 × 10^−5^)	(2.93 × 10^−5^)
Large jet/turbine IFR operations * 1–2 km	1.54 × 10^−5^	−5.85 × 10^−8^
	(4.72 × 10^−5^)	(3.05 × 10^−6^)
Small jet/turbine IFR operations * 1–2 km	7.68 × 10^−5^	7.39 × 10^−5^
	(9.37 × 10^−5^)	(5.83 × 10^−5^)
General aviation VFR operations * 1–2 km	−9.38 × 10^−7^	1.91 × 10^−6^
	(3.15 × 10^−6^)	(6.17 × 10^−6^)
Piston–engine IFR operations * 2–3 km	5.04 × 10^−5^ **	−2.31 × 10^−5^
	(2.28 × 10^−5^)	(1.93 × 10^−5^)
Large jet/turbine IFR operations * 2–3 km	4.18 × 10^−5^	2.22 × 10^−6^
	(6.63 × 10^−5^)	(1.56 × 10^−6^)
Small jet/turbine IFR operations * 2–3 km	−3.81 × 10^−6^	2.92 × 10^−6^
	(2.40 × 10^−6^)	(5.43 × 10^−6^)
General aviation VFR operations * 2–3 km	8.77 × 10^−5^ *	2.87 × 10^−5^
	(5.07 × 10^−5^)	(5.40 × 10^−5^)
Observations	6365	7880
Pseudo R2	0.189	0.146

(**a**) All models use CEM weights (calculated using a treatment definition consistent with the treatment group for each regression) and include block group fixed effects, year fixed effects, airport-year time trends, and all time-variant control variables shown in Table 2. Robust standard errors clustered by census tract are in parentheses. *** *p* < 0.01, ** *p* < 0.05, * *p* < 0.1. (**b**) This model uses CEM weights (calculated using the 3 km treatment group) and includes block group fixed effects, year fixed effects, airport-year time trends, and all time-variant control variables shown in Table 2. Robust standard errors clustered by census tract are in parentheses. *** *p* < 0.01, ** *p* < 0.05, * *p* < 0.1.

**Table 5:** Key coefficient results from single regression with 3 km treatment cutoff: Associations between airport proximity and wind direction and age 65+ cardiovascular mortality.

	Single-Runway Airports	Multi-Runway Airports
0–1 km	−0.196	−0.811 ***
	(0.208)	(0.307)
1–2 km	0.0407	0.137
	(0.101)	(0.111)
2–3 km	0.0589	−0.229
	(0.142)	(0.151)
0–1 km × downwind	5.839	9.177 **
	(6.173)	(4.191)
1–2 km × downwind	4.153	0.272
	(3.684)	(1.663)
2–3 km × downwind	2.564 *	−1.127
	(1.414)	(1.684)
Downwind	−1.231 **	0.137
	(0.508)	(0.867)
0–1 km × upwind	−5.000	0.943
	(5.108)	(2.124)
1–2 km × upwind	−5.858	−0.681
	(3.965)	(1.051)
2–3 km × upwind	−3.228	4.645 *
	(2.387)	(2.720)
Upwind	1.377	0.0401
	(1.246)	(0.568)
Observations	6365	7880
Pseudo R2	0.106	0.0703

This model uses CEM weights (calculated using the 3 km treatment group) and includes closest TFMSC airport fixed effects, year fixed effects, airport-year time trends, and time-variant and time-invariant control variables shown in Table 2. Robust standard errors clustered by closest airport are in parentheses. *** *p* < 0.01, ** *p* <0.05, * *p* < 0.1.

**Table 6:** Key coefficient results from single regression with 3 km treatment cutoff: Associations between annual piston-engine IFR operations and wind direction and age 65+ cardiovascular mortality.

	Single-Runway Airports	Multi-Runway Airports
Piston-engine IFR operations * 0–1 km	0.00107 ***	−0.000128
	(0.000373)	(0.000227)
Piston-engine IFR operations * 1–2 km	0.000103 ***	−6.89 × 10^−6^
	(3.76 × 10^−5^)	(4.18 × 10^−5^)
Piston-engine IFR operations * 2–3 km	5.44 × 10^−5^	−2.23 × 10^−5^
	(4.37 × 10^−5^)	(2.81 × 10^−5^)
Piston-engine IFR operations * 0–1 km × downwind	0.00356 ***	0.00156
	(0.00135)	(0.00149)
Piston-engine IFR operations * 1–2 km × downwind	0.000188	0.000173
	(0.000600)	(0.000251)
Piston-engine IFR operations * 2–3 km × downwind	−0.000431	−0.000192
	(0.000493)	(0.000284)
Piston-engine IFR operations * downwind	0.000295	−0.000134
	(0.000211)	(0.000162)
Piston-engine IFR operations * 0–1 km × upwind	−0.00656 ***	−0.000950
	(0.00190)	(0.000842)
Piston-engine IFR operations * 1–2 km × upwind	0.000942	2.57 × 10^−5^
	(0.000801)	(0.000491)
Piston-engine IFR operations * 2–3 km × upwind	0.000223	3.49 × 10^−5^
	(0.000458)	(0.000309)
Piston-engine IFR operations * upwind	−0.000487 **	−0.000372
	(0.000244)	(0.000229)
Observations	6365	7880
Pseudo R2	0.189	0.146

This model uses CEM weights (calculated using the 3 km treatment group) and includes block group fixed effects, year fixed effects, airport-year time trends, and all time-variant control variables shown in Table 2. Robust standard errors clustered by census tract are in parentheses. *** *p* < 0.01, ** *p* < 0.05, * *p* < 0.1.

## Data Availability

Mortality records from the North Carolina State Center for Health Statistics were obtained through an agreement with the Children’s Environmental Health Initiative (CEHI) at the University of Notre Dame. Readers who would like to use these data must apply to the North Carolina State Center for Health Statistics. Federal Aviation Administration (FAA) annual Airport Master Records (Form 5010) prior to 2020 were obtained by email communication with FAA. Readers who would like to use these data must contact the Federal Aviation Administration. The National Transportation Noise Map 2016 was obtained by email communication with the Bureau of Transportation Statistics. Readers who would like to use these data must contact the Bureau of Transportation Statistics. All other datasets used in this analysis are publicly available from various websites. Bureau of Labor Statistics. Local Area Unemployment Statistics: https://www.bls.gov/lau/#tables (accessed on 14 March 2021) Federal Aviation Administration (FAA). Traffic Flow Management System Counts (TFMSC): https://aspm.faa.gov/tfms/sys/main.asp (accessed on 8 April 2021) Federal Aviation Administration (FAA). Air Traffic Activity System (ATADS): https://aspm.faa.gov/opsnet/sys/airport.asp (accessed on 23 August 2021) Federal Aviation Administration (FAA). Airport Master Records (Form 2010) corresponding to current year. Airport Data and Information Portal: https://adip.faa.gov/agis/public/#/public (accessed 27 October 2020) IPUMS National Historical Geographic Information System: https://www.nhgis.org/ (accessed on 13 July 2021) National Oceanic and Atmospheric Administration. Climate Data Search: https://www.ncdc.noaa.gov/cdo-web/search (accessed on 28 March 2021) EPA RSEI Geographic Microdata (RSEI-GM): https://www.epa.gov/rsei/ways-get-rsei-results#microdata (accessed on 28 March 2021).

## Supplementary Materials

The following supporting information can be downloaded at: https://www.mdpi.com/article/10.3390/ijerph19105941/s1, Figure S1: Cardiovascular mortality rate (age 65 and older) by airport type and distance from airport: Full sample; Table S1: ICD-10 codes for cardiovascular deaths among individuals age 65 and older in North Carolina, 2000–2017; Table S2: Summary statistics by airport type and distance from airport: full sample; Table S3: Full coefficient results using single regression with a 3 km treatment cutoff: Association of airport proximity and cardiovascular mortality near TFMSC airports using CEM sample; Table S4: Key coefficient results from single regression model with a 4 km treatment cutoff: Impact of airport proximity on cardiovascular mortality within near TFMSC airports using full sample without matching; Table S5: Full coefficient results using single regression model with a 3 km treatment cutoff: Impact of annual flight operations on cardiovascular mortality near TFMSC airports using CEM sample; Table S6: Key coefficient results from single regression model: Impact of annual flight operations on cardiovascular mortality near TFMSC airports using full sample without matching; Table S7: Key coefficient results using single regression model with a 3 km treatment cutoff: Impact of only piston-engine annual flight operations on cardiovascular mortality near TFMSC airports.
