# Supplementary material for: Cardiovascular Mortality and Leaded Aviation Fuel: Evidence from Piston-Engine Air Traffic in North Carolina
Source: Int J Environ Res Public Health. 2022 May 13;19(10):5941. doi: 10.3390/ijerph19105941 (PMC9140422; doi:10.3390/ijerph19105941)
Supplement: Supplementary file 1 [file ijerph-19-05941-s001.zip › ijerph-1715911-supplementary.pdf]

Supplementary Materials

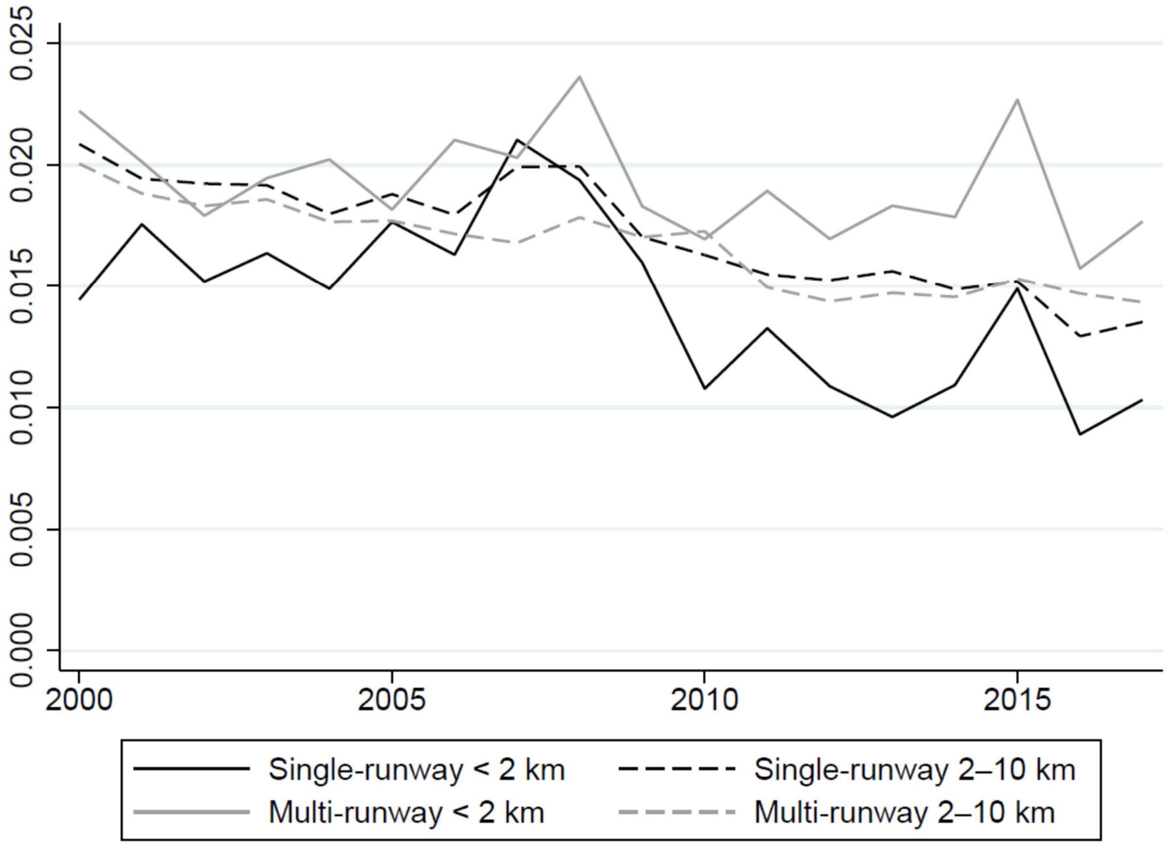

Figure S1. Cardiovascular mortality rate (age 65 and older) by airport type and distance from airport: Full sample.

Table S1. ICD-10 codes for cardiovascular deaths among individuals age 65 and older in North Carolina, 2000–2017.

	**Number of Deaths**	**Percentage of Deaths**
I00–I02—acute rheumatic fever	6	<1%
I05–I09—chronic rheumatic heart diseases	1161	<1%
I10–I15—hypertensive diseases	19,694	6%
I20–I25—ischemic heart diseases	144,190	45%
I26–I28—pulmonary heart disease and diseases of pulmonary circulation	5978	2%
I30–I52—other forms of heart disease	69,462	22%
I60–I69—cerebrovascular diseases	67,499	21%
I70–I79—diseases of arteries, arterioles and capillaries	12,171	4%
I80–I89—diseases of veins, lymphatic vessels and lymph nodes, not elsewhere classified	1042	<1%
I95–I99—other and unspecified disorders of the circulatory system	259	<1%
Total I00–I99—diseases of the circulatory system	321,445	100%

Table S2. Summary statistics by airport type and distance from airport: full sample.

	**Single-Runway Airports**		***p*-Value of Difference in Means**	**Multi-Runway Airports**		***p*-Value of Difference in Means**
	0–2 km	2–10 km		0–2 km	2–10 km	
Outcome variable						
Number of 65+ CVD deaths	2.48<br>(2.78)	3.13<br>(3.39)	<0.01	3.08<br>(3.4)	2.82<br>(3.18)	<0.01
Exposure variables						
Piston-engine IFR operations	2623.10<br>(2967.24)	3316.96<br>(3568.56)	<0.01	4247.64<br>(3809.28)	6983.97<br>(5935.27)	<0.01
Large jet/turbine IFR operations	2093.29<br>(5435.66)	2555.21<br>(5079.91)	<0.01	40,046.48<br>(115,189.7)	114,265.10<br>(178,902.1)	<0.01
Small jet/turbine operations	850.85<br>(1347.37)	1238.99<br>(1644.39)	<0.01	2332.06<br>(2201.26)	3856.31<br>(2896.38)	<0.01
General aviation VFR operations	27,550.65<br>(19,592.33)	30,504.71<br>(18,081.39)	<0.01	24,678.50<br>(11,730.21)	21,173.25<br>(10,955.67)	<0.01
Time-variant control variables						
65+ population	199.24<br>(143.84)	207.25<br>(158.41)	0.130	170.07<br>(105.96)	189.79<br>(143.35)	<0.01
Share Black population	0.08<br>(0.1)	0.18<br>(0.2)	<0.01	0.42<br>(0.31)	0.27<br>(0.26)	<0.01
Share Hispanic population	0.06<br>(0.07)	0.08<br>(0.1)	<0.01	0.08<br>(0.12)	0.07<br>(0.11)	0.02
	0.0004	0.0005	<0.01	0.0007	0.0008	<0.01

Population density	(0.0006)	(0.0005)		(0.0006)	(0.0007)	
Percent vacant housing	0.19 (0.21)	0.13 (0.14)	<0.01	0.13 (0.1)	0.11 (0.1)	<0.01
Percent rental housing	0.30 (0.21)	0.33 (0.23)	<0.01	0.49 (0.23)	0.41 (0.26)	<0.01
Median income (2010$)	50,844.89 (19,150.96)	47,704.57 (24,079.83)	<0.01	32,490.77 (14,485.4)	48,594.28 (26,628.13)	<0.01
Percent of adults 25+ with college degree	0.32 (0.2)	0.26 (0.2)	<0.01	0.17 (0.14)	0.31 (0.22)	<0.01
Percent pre-1950 housing	0.06 (0.07)	0.12 (0.14)	<0.01	0.17 (0.14)	0.13 (0.17)	<0.01
Days above 90 degrees	34.12 (24.63)	34.69 (24.97)	0.49	41.03 (22.54)	42.31 (21.97)	0.06
Unemployment rate	6.64 (2.67)	6.72 (2.62)	0.38	6.92 (2.56)	6.64 (2.49)	<0.01
Toxicity-weighted lead air concentration	1.88 (3.01)	3.12 (8.84)	<0.01	3.79 (7.32)	5.94 (29.38)	0.01
Toxicity-weighted total air concentration of chemical releases	5982.37 (37,304.68)	10,127.54 (56,802.82)	0.03	18,529.34 (76,438.31)	32,654.58 (177,830.3)	<0.01
Percent days downwind*	0.05 (0.03)	0.06 (0.04)	0.02	0.08 (0.03)	0.08 (0.03)	<0.01
Percent days upwind*	0.05 (0.03)	0.06 (0.04)	<0.01	0.07 (0.03)	0.08 (0.04)	0.02
Time-invariant variables (only included in airport fixed effects model)						
Percent > 55 decibel transportation noise	5.23 (6.15)	3.41 (2.9)	<0.01	9.07 (11.01)	5.17 (7.93)	<0.01
Heliport located within 2 km	0.04 (0.19)	0.07 (0.25)	<0.01	0.04 (0.21)	0.13 (0.33)	<0.01
Major road located within 2 km	0.96 (0.19)	0.85 (0.36)	<0.01	0.96 (0.21)	0.92 (0.27)	<0.01
Major road located within 500 m	0.20 (0.4)	0.30 (0.46)	<0.01	0.37 (0.48)	0.34 (0.47)	0.03
Hospital located within 2 km	0.06 (0.23)	0.10 (0.3)	<0.01	0.09 (0.29)	0.16 (0.37)	<0.01

Charlotte Motor Speedway located within 4 km × pre-2007 lead phaseout	0.01<br>(0.08)	0.00<br>(0.06)	0.11	0.00<br>(0)	0.00<br>(0)	-
N	968	12,774		1204	16,549	

Standard deviations in parentheses.

Table S3. Full coefficient results using single regression with a 3 km treatment cutoff: Association of airport proximity and cardiovascular mortality near TFMSC airports using CEM sample.

	**Single-Runway Airports**	**Multi-Runway Airports**
Located 0–1 km of TFMSC airport	−0.225 **	0.104
	(0.0942)	(0.128)
Located 1–2 km of TFMSC airport	−0.00456	0.108
	(0.0719)	(0.0752)
Located 2–3 km of TFMSC airport	0.0359	0.0132
	(0.0733)	(0.0706)
Share Black population	−0.0298	−0.0668
	(0.233)	(0.105)
Share Hispanic population	0.270	−0.304 **
	(0.183)	(0.143)
Population density	−71.21	−94.80 **
	(51.42)	(48.11)
Share vacant housing	0.322 *	−0.281
	(0.164)	(0.203)
Share rental housing	0.556 ***	0.495 ***
	(0.158)	(0.148)
Median income	1.27×10^{-6}	-2.90×10^{-6}
	(1.24×10^{-6})	(2.35×10^{-6})
Share college graduates	−0.703 ***	−0.137
	(0.231)	(0.207)
Share pre-1950 housing	0.147	0.237 *
	(0.281)	(0.128)
Days above 90 degrees	0.00135	0.000981
	(0.00132)	(0.00142)
Unemployment rate	0.0397 **	0.00675
	(0.0192)	(0.0165)
Toxicity-weighted lead air concentration	−0.000643	0.000118
	(0.00398)	(0.000137)
Toxicity-weighted total air concentration of chemical releases	3.24×10^{-7} **<br>(1.35×10^{-7})	-2.49×10^{-8}<br>(2.91×10^{-8})
Percent > 55 decibel transportation noise	−0.00347	−0.000526
	(0.00764)	(0.00185)
Heliport located within 2 km	0.203	−0.00313
	(0.124)	(0.0828)
Major road located within 2 km	0.227 ***	−0.0264
	(0.0688)	(0.0775)
Major road located within 500 m	0.149 **	0.00694
	(0.0751)	(0.0419)

Hospital located within 2 km	0.00807	0.226 ***
	(0.103)	(0.0656)
Charlotte Motor Speedway located within 4 km	0.106 **	
	(0.0528)	
Charlotte Motor Speedway located within 4 km * pre-2007 lead phaseout	−0.444 ***	
	(0.0387)	
General aviation VFR data missing		−0.0728 ***
		(0.0248)
Constant	−4.489 ***	−3.958 ***
	(0.206)	(0.167)
Observations	6365	7880
Pseudo R	0.104	0.0684

This model uses CEM weights derived based on a 3 km treatment cutoff and includes airport fixed effects, year fixed effects, and airport-year time trends. Robust standard errors clustered by closest airport are in parentheses. *** $p < 0.01$, ** $p < 0.05$, * $p < 0.1$.

Table S4. Key coefficient results from single regression model with a 4 km treatment cutoff: Impact of airport proximity on cardiovascular mortality within near TFMSC airports using **full sample without matching.**

	**Single-Runway Airports**	**Multi-Runway Airports**
0–1 km	−0.279 **	0.114
	(0.121)	(0.0696)
1–2 km	−0.0624	0.0734
	(0.0587)	(0.0637)
2–3 km	0.0171	−0.00935
	(0.0703)	(0.0625)
Observations	13,742	17,753
Pseudo R2	0.0957	0.0621

This model includes closest TFMSC airport fixed effects, year fixed effects, airport-year time trends, and control variables shown in Table 2. Robust standard errors clustered by closest airport are in parentheses. *** $p < 0.01$, ** $p < 0.05$, * $p < 0.1$.

Table S5. Full coefficient results using single regression model with a 3 km treatment cutoff: Impact of annual flight operations on cardiovascular mortality near TFMSC airports using CEM sample.

	**Single-Runway Airports**	**Multi-Runway Airports**
Piston-engine IFR operations*0–1 km	0.000857 **	1.26×10^{-5}
	(0.000361)	(5.78×10^{-5})
Large jet/turbine IFR operations*0–1 km	0.000282	5.50×10^{-5}
	(0.000195)	(0.000102)
Small jet/turbine IFR operations*0–1 km	-4.06×10^{-5}	4.99×10^{-5}
	(0.00110)	(0.000143)
General aviation VFR operations*0–1 km	-2.66×10^{-6}	-1.10×10^{-5}***

	(4.47×10^{-6})	(3.99×10^{-6})
Piston-engine IFR operations*1–2 km	0.000150 ***	1.22×10^{-5}
	(4.88×10^{-5})	(2.93×10^{-5})
Large jet/turbine IFR operations*1–2 km	1.54×10^{-5}	-5.85×10^{-8}
	(4.72×10^{-5})	(3.05×10^{-6})
Small jet/turbine IFR operations*1–2 km	7.68×10^{-5}	7.39×10^{-5}
	(9.37×10^{-5})	(5.83×10^{-5})
General aviation VFR operations*1–2 km	-9.38×10^{-7}	1.91×10^{-6}
	(3.15×10^{-6})	(6.17×10^{-6})
Piston-engine IFR operations*2–3 km	5.04×10^{-5} **	-2.31×10^{-5}
	(2.28×10^{-5})	(1.93×10^{-5})
Large jet/turbine IFR operations*2–3 km	4.18×10^{-5}	2.22×10^{-6}
	(6.63×10^{-5})	(1.56×10^{-6})
Small jet/turbine IFR operations*2–3 km	8.77×10^{-5} *	2.87×10^{-5}
	(5.07×10^{-5})	(5.40×10^{-5})
General aviation VFR operations*2–3 km	-3.81×10^{-6}	2.92×10^{-6}
	(2.40×10^{-6})	(5.43×10^{-6})
Piston-engine IFR operations at closest airport	-4.20×10^{-5} **	-1.10×10^{-6}
	(2.03×10^{-5})	(1.97×10^{-5})
Large jet/turbine IFR operations at closest airport	2.48×10^{-5}	-6.64×10^{-7}
	(2.60×10^{-5})	(1.78×10^{-6})
Small jet/turbine IFR operations at closest airport	-4.69×10^{-5}	1.36×10^{-5}
	(4.16×10^{-5})	(3.19×10^{-5})
General aviation VFR operations at closest airport	-9.37×10^{-7}	4.88×10^{-6}
	(2.37×10^{-6})	(3.61×10^{-6})
Share Black population	0.273	−0.0272
	(0.331)	(0.228)
Share Hispanic population	0.244	0.404
	(0.423)	(0.428)
Population density	−176.5	−326.2 ***
	(219.1)	(108.8)
Share vacant housing	0.759 **	0.261
	(0.352)	(0.299)
Share rental housing	0.276	0.785 ***
	(0.197)	(0.235)
Median income	2.26×10^{-6}	-2.20×10^{-6}
	(1.90×10^{-6})	(2.42×10^{-6})
Share college graduates	−0.123	0.0842
	(0.296)	(0.393)
Share pre-1950 housing	−0.235	−0.869
	(0.438)	(0.592)
Days above 90 degrees	0.00198	0.00186
	(0.00122)	(0.00149)
Unemployment rate	0.0352	0.00789
	(0.0226)	(0.0178)
Toxicity-weighted lead air concentration	0.00137	−0.000313
	(0.00214)	(0.000404)
Toxicity-weighted total air concentration of chemical releases	4.96×10^{-7} **	3.93×10^{-10}
	(2.17×10^{-7})	(6.60×10^{-8})
Charlotte Motor Speedway located within 4 km* pre-2007 lead phaseout	−0.492 ***	
	(0.116)	
General aviation VFR data missing		−0.00813

		(0.0681)
Constant	−3.716 ***	−4.162 ***
	(0.287)	(0.197)
Observations	6365	7880
Pseudo R2	0.189	0.146

This model uses CEM weights (derived based on a 3 km treatment cutoff) and includes block group fixed effects, year fixed effects, and airport-year time trends. Robust standard errors clustered by block group are in parentheses. *** $p < 0.01$, ** $p < 0.05$, * $p < 0.1$.

Table S6. Key coefficient results from single regression model: Impact of annual flight operations on cardiovascular mortality near TFMSC airports using **full sample without matching.**

	**Single-Runway Airports**	**Multi-Runway Airports**
Piston-engine IFR operations*0–1 km	0.000856 ***	-1.09×10^{-5}
	(0.000328)	(0.000101)
Large jet/turbine IFR operations*0–1 km	0.000409 **	3.79×10^{-5}
	(0.000174)	(6.44×10^{-5})
Small jet/turbine IFR operations*0–1 km	−0.000990	0.000145
	(0.000609)	(0.000103)
General aviation VFR operations*0–1 km	-6.91×10^{-6}	-9.91×10^{-6} *
	(6.20×10^{-6})	(5.89×10^{-6})
Piston-engine IFR operations*1–2 km	0.000115 ***	-2.49×10^{-5}
	(4.13×10^{-5})	(1.91×10^{-5})
Large jet/turbine IFR operations*1–2 km	-4.22×10^{-6}	-9.00×10^{-8}
	(3.58×10^{-5})	(1.82×10^{-6})
Small jet/turbine IFR operations*1–2 km	5.59×10^{-5}	3.73×10^{-5}
	(7.85×10^{-5})	(4.60×10^{-5})
General aviation VFR operations*1–2 km	-2.46×10^{-8}	2.04×10^{-6}
	(2.56×10^{-6})	(5.12×10^{-6})
Piston-engine IFR operations*2–3 km	3.34×10^{-5}	-2.06×10^{-5}
	(2.23×10^{-5})	(2.90×10^{-5})
Large jet/turbine IFR operations*2–3 km	3.42×10^{-5}	1.24×10^{-6}
	(6.07×10^{-5})	(3.57×10^{-6})
Small jet/turbine IFR operations*2–3 km	8.89×10^{-5} *	3.93×10^{-5}
	(4.72×10^{-5})	(5.13×10^{-5})
General aviation VFR operations*2–3 km	-4.40×10^{-6} ***	-7.82×10^{-7}
	(1.51×10^{-6})	(4.35×10^{-6})
Observations	13,742	17,753
Pseudo R2	0.178	0.154

This model includes block group fixed effects, year fixed effects, airport-year time trends, and all time-variant control variables shows in Table 2. Robust standard errors clustered by block group in parentheses. *** $p < 0.01$, ** $p < 0.05$, * $p < 0.1$.

Table S7: Key coefficient results using single regression model with a 3 km treatment cutoff: Impact of **only piston-engine** annual flight operations on cardiovascular mortality near TFMSC airports.

	**Single-Runway Airports**	**Multi-Runway Airports**
Piston-engine IFR operations * 0–1 km	0.000853 **	-4.40×10^{-5}
	(0.000337)	(8.43×10^{-5})
Piston-engine IFR operations * 1–2 km	0.000143 ***	1.39×10^{-5}
	(4.36×10^{-5})	(1.98×10^{-5})
Piston-engine IFR operations * 2–3 km	3.76×10^{-5}	-2.86×10^{-5}
	(2.52×10^{-5})	(1.76×10^{-5})
Observations	6365	7880
Pseudo R2	0.188	0.145

This model uses CEM weights (derived based on a 3 km treatment cutoff) and includes block group fixed effects, year fixed effects, and airport-year time trends. Robust standard errors clustered by block group are in parentheses. *** $p < 0.01$, ** $p < 0.05$, * $p < 0.1$.
